# Patients’ Perceptions of mHealth Apps: Meta-Ethnographic Review of Qualitative Studies

**DOI:** 10.2196/13817

**Published:** 2019-07-10

**Authors:** VanAnh Vo, Lola Auroy, Aline Sarradon-Eck

**Affiliations:** 1 Department of Epidemiology Columbia University New York, NY United States; 2 Université Grenoble Alpes, Centre National de la Recherche Scientifique, Sciences Po Grenoble, Pacte Grenoble France; 3 Aix-Marseille Université, Institut National de la Santé et de la Recherche Médicale, Institut de Recherche pour le Développement Sciences Economiques & Sociales de la Santé & Traitement de l'Information Médicale Marseille France; 4 Institut Paoli-Calmettes CanBios UMR1252 Marseille France

**Keywords:** mHealth, apps, mobile apps, qualitative studies, systematic review, mobile phone

## Abstract

**Background:**

Mobile phones and tablets are being increasingly integrated into the daily lives of many people worldwide. Mobile health (mHealth) apps have promising possibilities for optimizing health systems, improving care and health, and reducing health disparities. However, health care apps often seem to be underused after being downloaded.

**Objective:**

The aim of this paper is to reach a better understanding of people’s perceptions, beliefs, and experience of mHealth apps as well as to determine how highly they appreciate these tools.

**Methods:**

A systematic review was carried out on qualitative studies published in English, on patients’ perception of mHealth apps between January 2013 and June 2018. Data extracted from these articles were synthesized using a meta-ethnographic approach and an interpretative method.

**Results:**

A total of 356 articles were selected for screening, and 43 of them met the inclusion criteria. Most of the articles included populations inhabiting developed countries and were published during the last 2 years, and most of the apps on which they focused were designed to help patients with chronic diseases. In this review, we present the strengths and weaknesses of using mHealth apps from the patients’ point of view. The strengths can be categorized into two main aspects: engaging patients in their own health care and increasing patient empowerment. The weaknesses pointed out by the participants focus on four main topics: trustworthiness, appropriateness, personalization, and accessibility of these tools.

**Conclusions:**

Although many of the patients included in the studies reviewed considered mHealth apps as a useful complementary tool, some major problems arise in their optimal use, including the need for more closely tailored designs, the cost of these apps, the validity of the information delivered, and security and privacy issues. Many of these issues could be resolved with more support from health providers. In addition, it would be worth developing standards to ensure that these apps provide patients accurate evidence-based information.

## Introduction

Mobile health (mHealth) technology has been widely adopted in many countries worldwide. Since smartphones are already being used by many people, the latest technological innovations could improve access to health care, its delivery, and outcomes while decreasing the cost of health care by introducing evidence-based medical practices at the point of care and facilitating people’s access to medical information and data [1]. The health care industry should make use of these advantages by creating mHealth apps to improve patient care, as mentioned in the World Health Organization’s 2011 report [2]. The International Telecommunication Union estimates that by the end of 2018, there will be 107 mobile phone subscriptions per 100 inhabitants [3]. In addition to owning a mobile device, one-third of American smartphone users had downloaded health- and fitness-related apps onto their phones [4]. There are currently more than 100,000 health-related apps on the mobile phone app market, and according to the World Health Organization, 112 countries have reported the existence of at least one mHealth initiative [2].

mHealth apps are health apps available on a mobile device (a smartphone, tablet, or phablet), which can be used by both patients and their health care providers [5]. In a recent classification of mHealth apps, six main reasons were defined for using these apps: consulting medical information and references, communicating and/or sharing information, fulfilling a contextual need, obtaining educational tools, managing health professionals’ activities, and facilitating health-related management of patients [6]. It was reported that the introduction of mHealth technology in the health care industry was a slow process but that it is capable of revolutionizing health care, especially in developing countries [7]. However, health care apps seem to be frequently underused after being downloaded [8].

Since mobile phones and tablets are being increasingly integrated into the everyday lives of many people worldwide, mHealth apps provide some appealing possibilities for optimizing health systems, improving care and promoting health, and reducing health disparities. mHealth apps can provide patients with medical and health-related information (both general and personalized information) and education, improve patients’ compliance with treatment, and help them manage their own health (by conducting monitoring and diagnostic activities and improving their knowledge about their state of health or their illness). These promises explain why mHealth apps are frequently presented in the medical and public health literature as means of empowering patients [9].

Social scientists are more critical about the promise of increasing empowerment via mHealth. They have pointed out the existence of several moral and ethical issues associated with the emergence of these tools, such as the idea that the users of these technologies are ideal subjects who are responsible, self-disciplined, and motivated to improve their own health and mHealth’s intrusion into users’ private lives to record, survey, monitor, and discipline people [9]. Those taking a critical approach generally question whether mHealth practices may not be based on a rather consumerist vision of medicine in which patients’ relationship with care may tend to be based on the consumption of services, and patient-consumer satisfaction becomes the main issue [10].

Therefore, given the fast development and integration of mHealth apps, it has become imperative to document people’s perceptions, beliefs, and experience of mHealth apps as well as to determine how highly they appreciate them. Several academic papers have addressed the relevance of mHealth apps and solutions for dealing with a specific disease or state [5,11-14]. Other studies have addressed the implementation of electronic health (eHealth) from the physician’s perspective [15] or reviewed the evidence favoring the use of mobile technology by community health workers [16]. Based on a review of the quantitative surveys available in the literature, Azhar and Dhillon have modelled factors influencing the effective use of mHealth apps for self-care purposes [17]. However, since very little information is available on the patients’ perspective, the aim of this study was to review the latest findings on how patients perceive mHealth apps in order to establish whether they agree that the idea of prescribing apps more widely is potentially feasible and desirable.

## Methods

### Search Strategy

Using relevant electronic databases (PubMed and Web of Science), a systematic search was performed on the literature. Key concepts such as perception and experience of mHealth were used to search the databases. The search was completed using a Medical Subject Heading keyword combination (eg, “telemedicine” AND “qualitative study”) and other relevant keywords (Textbox 1).

The studies included in this review were related to mHealth or similar concepts (ie, telehealth apps, eHealth, or digital devices). Other terms and keywords used for this purpose were mobile health application(s) (or apps), eHealth app(s), telehealth devices, telehealth systems, and digital devices. Other keywords related to perception included in the search were experience, views, perspective, perception, feasibility, usability, review, utility, acceptability, evaluation, quantified self, and self-assessment. Keywords used to describe these apps were mHealth, mobile health, eHealth, telecare technologies, apps, mobile health, health technology, mobile applications, smartphones, digital health, telemedicine, and mobile apps. We restricted our focus to one main population of users, consisting of patients and potential patients. In order to determine what patients believe and how they perceive mHealth apps, we were particularly interested in original studies using a qualitative approach. Qualitative methods are potentially useful for understanding the individual needs of patients, their experiences, and their perception of mHealth apps [18]. Combinations of keywords including the term “qualitative study” were also used. In addition to the results obtained by searching databases and journals, other references to relevant articles were retrieved by performing a manual search. The studies included had to be in English and had to have been published within the last 5 years (from January 2013 to June 2018). The use of smartphones, especially iPhones and Androids, increased sharply to over 55% in 2013, along with the use of smartphone apps [19]. We therefore decided to focus on articles published within the last 5 years.

**Concept and keywords used in the search strategy.**

**Device related:**

- Digital devices
- Medical app(s)
- mHealth
- mHealth app(s)
- Mobile health
- Smartphone app(s)
- Telecare technologies
- Telemedicine

**Perception/value/belief related:**

- Acceptability
- Evaluation
- Experience
- Feasibility
- Perception
- Perspectives
- Review
- Usability
- Utility

**Type of study:**

- Qualitative
- Qualitative study
- Literature review

**User:**

- Patients
- Physicians
- Providers

### Inclusion and Exclusion Criteria and Quality Assessment

Since this research project focused on how mHealth apps can be used to improve patients’ health care, any apps designed for the sole purpose of surveillance, location tracking, consultation, changing health behavior or health styles, or monitoring patient activity were excluded from the study. Since the focus was also restricted to patients, any studies focusing only on caregivers and other members of patients’ social networks (such as spouses and parents) were excluded. Lastly, papers only reviewing an app’s user interface and usability were not included unless they contributed to understanding patients’ perception of mHealth app usage.

In view of the fast progress of technology, papers related solely to the use of short message service were not included because information, reminders, and other data are communicated via mHealth apps themselves. Studies on the wide range of digital and other technological tools that are in development, such as FitBits, eHealth, telemonitoring devices, and telehealth systems, were also excluded in order to focus on mobile phone apps alone.

Since this review examines studies with qualitative designs, all relevant articles were finally double checked to make sure that they were in line with the Consolidated Criteria for Reporting Qualitative Research, after which no further studies were excluded.

### Data Extraction and Analysis

The titles and abstracts were scanned to retrieve the keywords and combinations of keywords mentioned above in order to identify relevant articles and exclude those that were not within the scope of this review. The Methods section of each article was reviewed extensively to ensure that each study was based on a qualitative design. Full texts of relevant articles were retrieved and further reviewed to ensure that they matched the objectives of this study, and any incomplete studies and studies in progress were excluded. To list the information presented in each paper, a table was drawn up on an Excel file, in which the following details were recorded: author, country involved in the study, study population and demographics, methods, disease/condition of interest, type and purpose of the app, results, themes, and time scheme.

Data were extracted from the articles included in this review using the meta-ethnographic framework and the corresponding interpretative method, which was designed for developing new interpretations via comparisons rather than aggregate findings [20]. The first step consisted of arranging these studies in chronological order and extracting the themes from the Results and Discussion sections. While continuing to analyze other articles, we continued to keep an eye out for any emerging themes and include them in the ongoing analysis.

## Results

### Study Selection Procedure

**Figure 1:**
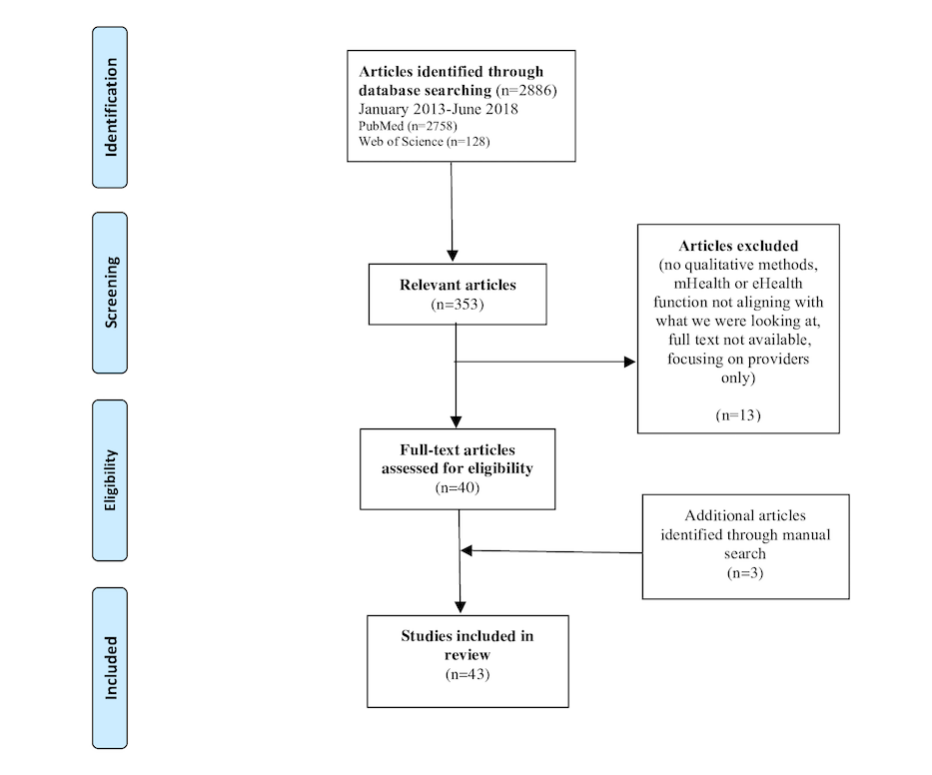
Preferred Reporting Items for Systematic Reviews and Meta-Analyses flow diagram of study selection.

The search conducted on the literature yielded 356 articles, 43 of which met the inclusion criteria (Figure 1). Three of these articles were included as the result of a manual search, while the other 40 articles were found by consulting databases. The reference numbers, the authors’ names, the year of publication, the countries in which the studies were conducted, data on participants and their disease/condition, the methods used, the purpose of the app/device, and the most significant findings obtained in the studies selected are all presented in Table 1.

**Table 1:** Characteristics of the selected studies.

Study	Country	Methods and participants	Disease/condition	Type of device/app	Purpose of device/app	Evaluation or expectation of app	Results
Hilliard et al, 2014 [21]	US	Questionnaires and telephone interviews; patients (n=16)	Cystic fibrosis	mHealth^a^ app	Treatment adherence, disease management	Evaluation after use of app	Benefits: access to information, socialization with the cystic fibrosis community, enhance communication with the health care team, support prescription refills Critiques: apps need to support those with cystic fibrosis, so they must be customized
Lubberding et al, 2015 [22]	The Netherlands	Face-to-face interviews; patients (n=30)	Head and neck, breast cancer	eHealth^b^ app	Monitors quality of life, gives advice/feedback and referrals	Expectation of eHealth app	Survivors determined that the eHealth app could be valuable for follow-up of cancer care by enabling them to monitor quality of life, personalized advice, and supportive care
Schnall et al, 2015 [23]	US	Focus groups; patients (n=50)	HIV	mHealth app	Management and prevention of HIV (via adherence and retention of HIV medication)	Evaluation after use of app	Benefits: empowers with the sense of autonomy and helped patients in their decision making, increases competency in self-management and sense of belonging and attachment
Schnall et al, 2015 [24]	US	Focus groups; providers (n=30) and patients (n=50)	HIV	mHealth app	Monitoring and managing health of people living with HIV/AIDS, communication with providers	Evaluation after use of app	Benefits: potentially useful, can facilitate delivery of care, and helps self-manage Critiques: security and privacy of app, need an app that is simple and easy to understand
Martinez, 2015 [25]	US	Focus groups; patients (n=27)	Chronic disease	mHealth app	Patient attitudes toward mHealth technology to best tailor interventions to the needs of high-risk adults patients living with chronic disease	Expectation of eHealth app	Benefits: quick communication with health care providers, self-monitoring, self-management Critiques: confidentiality, security, expenses, customizability, depersonalized interactions with medical community
Egsgaard et al, 2016 [26]	Denmark	Questionnaires and face-to-face interviews; patients (n=82)	Chronic pain	Tablet app	Identifying location of pain	Evaluation after use of app	App helped patients accurately describe and locate pain
Israni et al, 2016 [27]	US	Face-to-face interviews; patients (n=16)	Kidney	mHealth app	Medication adherence	Expectation of app	Kidney transplant recipients responded positively on the potential app specifically for their condition Critiques: concern about technical details, need to include a few features for their condition
Knight et al, 2016 [28]	Australia	Focus groups; patients (n=7)	Diabetes	mHealth app	Diabetes bolus calculator for medication	Evaluation after use of app	Benefits: useful self-management tool, improves usability Critiques: low health literacy
Lupton, 2016 [29]	Australia	Focus groups; patients (n=36)	Pregnancy	mHealth app	Access to information on pregnancy	Evaluation after use of app	Benefits: detailed and immediate information, entertainment, facilitates communication and socialization, reassuring
Puszkiewicz et al, 2016 [30]	UK	Questionnaires and telephone interviews; patients (n=11)	Cancer	mHealth app	Promotion and management of physical activity in cancer survivors	Evaluation after use of app	Benefits: helpful exercise instructions, did not cause any injuries or specific problems Critiques: need to be personalized for cancer survivors in terms of lifestyle and fitness needs, add in feature on socialization
Rosen et al, 2016 [31]	US	Focus groups; patients (n=22)	HIV	mHealth app	Management for medication adherence and CD4/viral load counts	Evaluation after use of app	Benefits: assists in adhering to drug regimen Critiques: notifications are too frequent, privacy and security, requests too much information
Simons et al, 2016 [32]	UK	Focus groups; providers (n=31), parents (n=9), and patients (n=19)	Attention deficit hyperactivity disorder	mHealth app	Collection of physiological/health-related data	Evaluation after use of app	Benefits: improved and supported management of attention deficit hyperactivity disorder in between appointments, improved quality of appointments, supported self-management Critiques: burden on clinics, privacy and confidentiality issues of data, credibility and validity of sources
Young-Afat et al, 2016 [33]	The Netherlands	Face-to-face interviews; providers (n=10) and patients (n=15)	Breast cancer	mHealth app	Collection of patient-reported outcomes of breast cancer patients	Evaluation after use of app	Benefits: potentially be in more control of health Critiques: does not provide anything more than medical team and internet
Bendixen et al, 2017 [34]	US	Focus groups; providers (n=11) and patients (n=16)	Brain and spinal cord anomalies	mHealth app	Self-management	Evaluation after use of app	Benefits: engaging, add value to daily life, accessible information, relevant to health needs Critiques: add feature for socialization and make it customizable
Cai et al, 2017 [35]	UK	Face-to-face interviews and focus groups; providers (n=21), parents (n=7), and patients (n=29)	Juvenile idiopathic arthritis	mHealth app	Monitor symptoms and facilitate engagement with providers and patients	Evaluation after use of app	Benefits: high levels of acceptability and usability, can improve health care and outcomes
Fleming et al, 2017 [36]	US	Face-to-face interviews; patients (n=9)	Mental health	mHealth app	Management of anxiety and depressive symptoms	Evaluation after use of app	Benefits: facilitate engagement with patient and provider Critiques: app needs to be culturally tailored for young sexual minority men
Goetz et al, 2017 [37]	Germany	Face-to-face interviews; patients (n=30)	Pregnancy	eHealth and mHealth app	Patient engagement of clinical routine care/pregnancy care	Evaluation after use of app	Benefits: facilitates socialization with other mothers and providers, easy and quick access to information, overall positive attitude toward using eHealth app Critiques: need for personalization, lack of scientifically validated sources, add feature for immediate feedback, data security
Hälleberg Nyman et al, 2017 [38]	Sweden	Face-to-face interviews; patients (n=28)	Prostate cancer	mHealth app	Management, reporting of symptoms during radiotherapy for patients with prostate cancer, symptom and risk assessment, alerts via SMS^c^	Evaluation after use of app	Benefits: facilitates conversation between patient and provider
Huerta-Ramos et al, 2017 [39]	Spain	Focus group and face-to-face interviews; providers (n=13), family members (n=9), and patients (n=14)	Schizophrenia	mHealth app	Empowerment, individualizing treatment and improving understanding of the illness	Evaluation after use of app	Benefits: access to reliable information regarding disease and support, improved contact with clinicians, support of self-management of daily tasks and appointments Critiques: wanted more human contact with clinicians
Langius-Eklöf et al, 2017 [40]	Sweden	Face-to-face interviews; patients (n=66)	Prostate cancer	mHealth app	Manage symptoms from radiotherapy for prostate cancer patients, risk assessment, alerts via SMS to providers, access to information	Evaluation after use of app	Benefits: app is easy and efficient to use, increased security and well-being, improved self-management
Mistler et al, 2017 [41]	US	Questionnaire and face-to-face interviews; patients (n=13)	Mental health	mHealth app	Self-management of and treatment	Evaluation after use of app	Benefits: app is easy to use, relieved anxiety, sleep, and boredom
Nightingale et al, 2017 [42]	UK	Face-to-face interviews and focus groups; providers (n=7), parents (n=12), and patients (n=12)	Chronic kidney disease	mHealth app	Management of treatment and dietary regiments, treatment adherence	Evaluation after use of app	Expectation: accessible information, engaging/interactive and developmentally appropriate care-management app, endorsement from renal professionals, supplementary to professionals
Sebern et al, 2017 [43]	US	Focus groups; providers (n=7) and patients (n=8)	Heart failure	mHealth app	Education and self-management, monitor symptoms and physical activity for patients of heart failure	Evaluation after use of app	Benefits: facilitates self-management and communication Critiques: cost, overwhelming
Velu et al, 2017 [44]	The Netherlands	Focus groups; providers (n=12) and patients (n=2)	Obstetric care	mHealth app	Assess health risk of working pregnant women	Evaluation after use of app	Benefits: accessible to practical and understandable information Critiques: extensive battery and memory use, notifications too frequent
Westergaard et al, 2017 [45]	US	Face-to-face interviews; patients (n=19)	HIV	mHealth app	Medication adherence/management, monitor risk behaviors of patients with substance use and HIV	Evaluation after use of app	Benefits: manage HIV care when busy or stressed, empowered them to support others, socialization
Webb et al, 2017 [46]	Australia	Questionnaires and phone interviews; patients (n=14)	Mental health	mHealth app	Health and lifestyle screening tool	Evaluation after use of app	Benefits: using app allowed participants to easily disclose sensitive issues raised in their consultation, participants felt more prepared and in control
Bauer et al, 2018 [47]	US	Questionnaires and face-to-face interviews; patients (n=17)	Mental health	mHealth app	Symptom monitoring, self-management of mental health, connect with collaborative care program	Evaluation after use of app	Benefits: facilitated discussion, supported relationship between patient and providers Critiques: lack of personalization, privacy and data security
Cordova et al, 2018 [48]	US	Questionnaires and face-to-face interviews and focus groups; patients (n=30)	HIV	mHealth app	HIV intervention app	Evaluation after use of app	Benefits: facilitate adolescent-clinician communication, engaging and informative, interesting (culturally fitting to adolescents) Critiques: Confidentiality of risk assessment
Dahlberg et al, 2018 [49]	Sweden	Face-to-face interviews; patients (n=18)	Postoperative recovery	eHealth app	Assess and follow-up on postoperative recovery day after surgery	Evaluation after use of app	Benefits: supportive, informative, facilitates communication, socialization
Desveaux et al, 2018 [50]	Canada	Telephone interviews; patients (n=16)	Type II diabetes	mHealth app	Management and adherence to insulin for patients with type II diabetes	Evaluation after use of app	Benefits: supports self-management, increase awareness Critiques needs specific feedback, includes feature that acknowledges and recognizes successes, time consuming
Floch et al, 2018 [51]	European countries	Face-to-face interviews and netnography; providers (n=33), parents (n=17), and patients (n=24)	Cystic fibrosis	mHealth app	Access to information, manage treatment and follow-up	Expectation before use of the app and evaluation after use of the app	Critiques: needs an app that is easy to use, customizable, and will support self-management; takes into account the needs of individuals with cystic fibrosis and their busy personal life
Giunti et al, 2018 [52]	Switzerland	Questionnaires, focus group, and face-to-face interviews; providers (n=12) and patients (n=12)	Multiple sclerosis	mHealth app	Health promotion	Evaluation after use of app	Benefits: realistic and positive feedback, minimize usability burdens Critiques: design, validity of information, need to emphasize that app is secondary to provider, need to be more engaging (eg, games), personalization, health literacy, privacy and data ownership, socialization
Grist et al, 2018 [53]	UK	Face-to-face interviews; patients (n=40)	Self-harm/mental health	mHealth app	Management of those who self-injure by tracking moods, promoting mood changing activities, etc	Evaluation after use of app	Benefits: helpful in managing their condition, privacy and discreetness of app, easy to use, Critiques: poor personalization
Hirschey et al, 2018 [54]	US	Questionnaire and face-to-face interviews; patients (n=12)	Atrial fibrillation	mHealth app	Self-care and treatment adherence for patients with atrial fibrillation who are prescribed NOACs^d^	Evaluation after use of app	Benefits: easy to use, supported self-care and treatment adherence, information accessible
Husted et al, 2018 [55]	Denmark	Face-to-face interviews; patients (n=20)	Type 1 diabetes mellitus	mHealth	Management/adherence	Evaluation after use of app	Benefits: socialization, sense of competence safety, empowered to ask for help Critiques: lack of motivation for long-term app use
Jibb et al, 2018 [56]	Canada	Telephone interviews; patients (n=20)	Cancer	mHealth app	Pain management support	Evaluation after use of app	Benefits: supported self-management, engaging, facilitates discussion with provider Critiques: notifications too frequent, technical problems
Morrissey et al, 2018 [57]	Ireland	Focus groups; patients (n=24)	Hypertension	mHealth app	Medication adherence, management of hypertension	Evaluation after use of app	Benefits: motivates engagement with physician and self-management Critiques: using an app meant acknowledgment of the disease/condition or failure of memory, app is too challenging to use, app should include medical care in case of emergency, data regulation, cost of tools that is needed to use the app, burden of reminders
Riis et al, 2018 [58]	Denmark	Questionnaires and face-to-face interviews; patients (n=15)	Lower back pain	eHealth app	Access to information and advice for those with lower back pain	Evaluation after use of app	Benefits: self-management, informative Critiques: difficult to navigate, people prefer to speak to provide, need support from providers to use app, information should be presented comprehensively and succinctly, customization
Shorey et al, 2018 [59]	Singapore	Face-to-face interviews; patients (n=17)	Pregnancy	mHealth app	Access to health care information for postnatal care	Evaluation after use of app	Benefits: convenient, empowering, made providers more accessible, socialization, emotionally supported Critiques: technical issues, extend duration of use, provide more information
Switsers et al, 2018 [60]	Belgium	Focus groups; patients (n=16)	Bipolar disorder	mHealth app	Self-management of bipolar disorder	Perception of mobile health apps prior to usage	Benefits: self-management, informative, socialization Critiques: customization, frequency of feedbacks
Waite-Jones et al, 2018 [61]	UK	Focus group and face-to-face interviews; providers (n=8), parents (n=8), and patients (n=9)	Juvenile arthritis	mHealth app	Self-management of juvenile arthritis, access to information and self-management strategies	Evaluation after use of app	Benefits: facilitates self-management, informative Critiques: customization, security, add more functions to app (notifications, mindfulness/relaxation techniques, gamification), provide age/gender relevant information, security, cost
Anstey Watkins et al, 2018 [62]	South Africa	Face-to-face interviews; providers (n=43) and patients (n=113)	Chronic diseases	mHealth app	Information and service regarding chronic disease, pregnancy	Expectations prior to app use	Benefits: facilitated management of treatment, accessible information Critiques: limited functions on their phones, cost
Zhu et al, 2018 [63]	China	Face-to-face interviews; patients (n=13)	Breast cancer	mHealth app	Access to education/information on breast cancer, facilitating communication with peers and providers, symptom management	Evaluation after use of app	Benefits: accessible information, facilitates management of condition, empowering in confidence and emotional well-being, easy and convenient to use Critiques: technical difficulties, make language succinct and comprehensible, information app provides should be updated regularly, quick feedback

^a^mHealth: mobile health.

^b^eHealth: electronic health.

^c^SMS: short message service.

^d^NOAC: Nonvitamin K antagonist oral anticoagulant.

In 31 studies, the participants were all patients, whereas in 12 studies, the participants included both patients and providers. All 43 articles discussed patients’ opinions about including patient-centered mHealth apps or eHealth apps in their mobile phones. The aim of 37 of the 43 articles was to report patients’ assessment of an app, while 5 articles included either patients’ expectations or their perception of an app prior to its use, and one study included both their expectations and assessments.

Most of the articles reviewed included populations inhabiting developed countries, apart from one that focused on a developing country (South Africa). Most of these qualitative studies focused on the United States [21,23-25,27,31,34,36,41,43,45,47,48,54] and the United Kingdom [30,32,35,42,53,61]. Other countries included were the Netherlands [22,33,44], Denmark [26,55,58], Sweden [38,40,49], Australia [28,29,46], Canada [50,56], South Africa [62], Singapore [59], China [63], Germany [37], Ireland [57], Belgium [60], Spain [39], and Switzerland [52]. One paper included populations originating from seven European countries [51]. Although all the articles selected had been published during the last 5 years, about 70% of them were published more recently, during the last 2 years (2018: n=17; 2017: n=12). In addition, 8 articles were published in 2016, 4 articles were published in 2015, and 1 article was published in 2014.

Most of the apps under consideration were tailored to deal with chronic diseases: cancer [22,30,33,38,40,56,63]; HIV [23,24,31,45,48]; diabetes [28,50,55]; hypertension and cardiovascular diseases [43,54,57]; chronic kidney disease [27,42]; cystic fibrosis [21,51]; chronic pain [26,58]; juvenile arthritis [35,61]; brain and spinal cord anomalies [34]; multiple sclerosis [52]; chronic illnesses, in general [25,62]; and mental health disorders [32,36,39,41,46,47,53,60]. Other apps were specific to pregnancy/obstetric care [29,37,44,59], and one was for postoperative care [49]. Since almost all the studies’ objectives focused on chronic diseases/conditions, the views and expectations of patient users were related to apps designed for the purpose of providing support, including giving access to information, promoting interventions, promoting compliance with treatment, assisting with the management of treatment or disease, and facilitating discussions.

This article discusses the main strengths and weaknesses of using mHealth apps from the patients’ point of view. The strengths mentioned can be categorized into two main aspects: patients’ engagement and their empowerment. The four main weaknesses to which the subjects objected were the apps’ lack of trustworthiness, appropriateness, personalization, and accessibility (Textbox 2).

### Increasing Patient Engagement

#### Improving the Accessibility to Information

Apps are frequently used to make information accessible to users. In many cases, the apps under consideration here included information about a specific disease or condition or the medication available, to help patients handle their situation. Other apps were designed to assist patients by explaining their medication and medical treatment regimens. For the more educational apps, patients expressed their appreciation of the possibility of increasing their knowledge about health topics related to their disease or condition. However, some patients, such as cancer survivors, do not want more information and advice than they have already received at the hospital, because receiving more information might increase their anxiety [22].

#### Facilitating Two-Way Communication With Health Care Providers

Patients described mHealth apps as tools facilitating discussions with their providers. Making it possible to communicate easily with providers seems to be one of the great promises of mHealth [25]; some tools include a function that enables patients to contact their providers to ask questions or express concerns. For example, young individuals with diabetes who were interviewed by Husted et al [55] said they had experienced greater continuity in their patient-health care provider relationships and were more highly motivated to improve their self-management of diabetes, since their health care providers immediately responded to the questions they asked in the chat room. Some of the patients who were able to use a mobile phone app efficiently stated that these tools enhanced their experience of the health care services [38]. One patient reported that they made it possible to personalize their messages to their provider or health care professional and receive useful responses. The mobile phone device was described as a tool for patients to have a “two-way dialogue” with their health care professionals, making it “a security line and […] a link to someone who was caring for you and being in control of the situation” [38].

**Summary of the emerging themes.**

**Strengths mentioned by patients:**

- Engaging patients more strongly
  - Improving the accessibility of information
  - Facilitating two-way communication with health care providers
  - Peer support

- Increasing patient empowerment
  - Facilitating self-management
  - Gaining greater control and autonomy

**Weaknesses mentioned by patients:**

- Concerns of trustworthiness
  - Scientific validity
  - Technical validity

- Appropriateness as an essential quality
  - Relevance to specific diseases and conditions
  - Cultural and user appropriateness
- The need for greater personalization
- Accessibility issues

Patients also mentioned that these tools make for pleasant participation on the part of both users and health care professionals. They also described it as “passive receipt of care,” which was readily accepted by patients. With this type of care, they were able to accept and “resign themselves to receiving care without taking up the possibility to engage in active participation” [38].

Patients also felt that with the knowledge they had acquired, they were able to address topics they previously thought to be unimportant or irrelevant. The new information they received about what to bring into discussions with providers may enhance patients’ engagement with their providers. For example, patients using an app to describe their pain found that this helped them collaborate and interact with their physicians more effectively than using verbal descriptions and physical gestures. In addition, patients prefer gender-specific 3D body charts giving a “detail[ed] and realistic representation of the body” [26]. Patients described this body chart app as a “tool to facilitate communication of pain,” which is likely to “lead to improvements in pain communication, and thus facilitate clinical reasoning and treatment strategies” [26]. This will therefore help providers make more accurate assessments and more appropriate treatment plans for their patients.

However, it is worth noting that although the patients interviewed felt they had become more engaged in self-management owing to the use of these apps, they stressed the fact that apps should be used only as a complementary tool. Although mHealth apps can change the dynamics of patient-provider relationships by providing relevant information for making assessments, diagnoses, prescribing treatment, and so forth, patients prefer to use them simply as tools for facilitating these relationships and not for replacing them. Users believe apps to be a positive addition to the clinical process only, as one participant in the study by Simons et al [32] said, it “adds to what’s there already...not if it’s used as an excuse to see people less.” Apps could also be used to fill the gap between two doctors’ follow-up sessions [59]. On the other hand, some elderly patients fear that mHealth may lead to more depersonalized interactions with their doctors [25].

#### Peer Support

Not only were patients able to engage with their providers more effectively, but they also stated that some apps facilitated conversation with other people who had undergone similar experiences, via forums or chat rooms. Some apps also enabled patients to interact more easily with caregivers and other members of their social network. By facilitating exchanges between patients and other individuals, these apps enhance patient socialization by alleviating their social isolation and providing social support. Providing peer-to-peer support by sharing feelings, practical knowledge, and experience was found to be the main benefit of apps with a chat room for young people with diabetes [55]. It has been stressed that the process of socialization achieved by chatting with other people who are experiencing a similar situation is one of the most helpful aspects of an educational app for new parents during the postnatal period [59]. The users of this app were parents of newborn infants who found it comforting that they were able to link up with other new parents who were “linkable” and “going through the same things,” which results in parents providing answers that are directly relevant to the question [59]. However, not everyone is comfortable chatting on social networks. Some patients, especially those with cystic fibrosis, fear that comparing oneself with other people may lead to negative feelings or discouragement [21]. On the whole, mHealth apps can play a social role by improving patient self-management of their disease-related and other health-related issues [23].

### Increasing Patient Empowerment

#### Facilitating Self-Management

Many patients approve of apps that can be used for self-monitoring and self-management of their health [25]. In addition, it is worth noting that patients who were high engagers in self-management described the use of mHealth apps as beneficial and not a barrier or an interference. High engagers were “interested in using mobile technology to improve their health and enthusiastically engaged with the app immediately and consistently thereafter” [50]. Among moderate users, these tools were perceived as useful means of increasing their awareness of their actions and keeping track of the details in the management of their disease, whereas those who had little to no self-involvement in the management of their health were skeptical about using mHealth apps as a means of self-management. The level of engagement of the members of this group was found to be minimal to low. They were not able to integrate these apps successfully into their daily routine and stated that they were a burden. Overall, the perceived value of these apps and their integration as well as patients’ engagement with the tool depend on how self-reliant the patients are.

#### Gaining Greater Control and Autonomy

Patients provided with a tool that gave them access to useful supplementary information and helped them engage with their providers declared that they felt more empowered and in control of their condition, disease, or regimen. When the information provided in the app is succinct, comprehensible, and easily accessible, patients feel that they can improve their knowledge about their disease or condition, symptoms, and medication. One participant noted that one of the apps had too much content, and she felt “overwhelmed by the information each time [she] opened it. [She did] not have the patience to read all of [it]...” [63]. Therefore, in order to ensure that patients will engage fully with an app, the information provided must be clearly and concisely presented in laymen’s terms [58].

In addition to the supplementary information provided by these apps, the possibility of recording treatment-related conversations with physicians or their medical team gives patients a sense of control, especially when they are distressed [33]. Patients also mentioned that it would be beneficial to be able to edit and share the files recorded. However, recordings of meetings and consultations would have to be hashed out in detail to make sure that patients’ identity and data are protected.

Apps provide young people with chronic conditions a sense of autonomy and help develop skills such as problem-solving and decision-making skills and the ability to use resources and build relationships with professionals [61]. Having a tool equipped with these functions provided them with a “secure and supportive environment” [61]. Young patients often stated that having an interactive app with which they were in control provided them with information, a means of monitoring symptoms, and social support [61]. Patients found self-monitoring apps to be especially helpful for managing their symptoms or their mood in the case of bipolar disorders, by providing personalized feedback [32,60]. With self-monitoring apps, patients are able to gain control over their own health and health care and feel more empowered when consulting a doctor. They are usually enthusiastic about apps and willing to engage with them. However, some other patients tend to feel that apps are too demanding, which leads them to give up on using them regularly [57]. Apps should not have too many push notifications, as they can overwhelm or burden the users.

In short, with their newly acquired knowledge, patients felt they were well equipped to manage their own disease or condition, symptoms, medication, health-related behavior, and test results, thus facilitating the decision-making process and making them feel reassured and empowered.

### Concerns of Trustworthiness

#### Scientific Validity

Most of the patients interviewed said they were in agreement with the concerns sometimes expressed about the validity of the information provided on mHealth apps. Although patients found mHealth apps informative and supportive, they also expressed worry about how reliable the information may be. Patients mentioned that without the basic support they received from their providers, they would be much less inclined to trust the information provided by apps. Patients are sometimes rather skeptical of their apps because there is no proof that the information provided is evidence based or obtained from a reliable source. They suggested that it would be better if providers were able to back up the information conveyed by apps. They suggested that app designers should use evidence-based information and cite the sources of the information conveyed in order to confirm its validity. As mentioned in Goetz’s study [37], pregnant women, in particular, specified the need for scientifically valid data. Many of the users of these apps objected that the quality of the information they provide is questionable. Patients even explicitly stated that if their provider recommended an app, “it would make a difference” [58]. Having an app recommended by a physician familiar with evidence-based information is one of the most important criteria according to the users.

#### Technical Validity

The main issues mentioned by users in connection with these apps were those of privacy and security. Many of these apps ask users for sensitive information to achieve optimum performances. Some apps also allow providers to send patients personal data and findings via the app. Patients were concerned about the security of the apps, how many parties were able to view their data, and whether a data breach might occur. They wanted to know what the consequences of a situation of this kind might be and how it could be dealt with.

Concerns about privacy and data ownership were voiced in Giunti’s study [52], in which patients said they were not happy about having a third party, such as pharmaceutical or insurance companies, having access to their data. One patient put this point quite clearly: “If everyone could see my data, I wouldn’t give [the app] a chance” [52]. Similar findings were obtained in connection with an app that was tailored for HIV patients [24]. Patients expressed great concern about the privacy of their information, how much personal information they were willing to contribute, and the risk of being tracked [24,31]. Participants in the study by Martinez [25] mentioned “hackers” and “big brother” “to express their mistrust regarding the current state of digital information security” [25].

### Appropriateness as an Essential Quality

#### Relevance to Specific Diseases and Conditions

Several articles reviewed reported that many patients interviewed reported that these apps should be designed more closely in line with their condition or their disease, keeping individual users’ lifestyles and needs in mind. For example, since patients with cystic fibrosis have to follow a very strict multidrug treatment regimen, using an mHealth app could help them comply with it [51]. However, many patients find that the apps designed to promote compliance with drug treatment for cystic fibrosis did not take into account the busy lives these patients lead. Frequent alarms and notifications to take their medication may be annoying and consume their phone battery. Patients would prefer apps to be customizable to suit the user and tailored to meet individual preferences [25]. For example, there should be a “snooze” option if the medication cannot be taken immediately, or it should be possible to personalize the function so that their multiple-drug intake can be recorded in their schedule [21].

#### Cultural and User Appropriateness

Patients also stressed the need for apps to understand their users. It is essential for app developers to know who their end users are going to be and to ensure that the products are appropriate for them. For example, an HIV management app tailored for young men who have sex with other young men should be tailored to the specificities of this population. In this particular case, the author of the study mentioned that these young male patients would like to have an app that is colorful, bright, and visually appealing [36]. Developers need to keep the users and users’ culture in mind in order to ensure their engagement [48]. Young people usually prefer youth-friendly language and designs [35,46,61].

Patients expressed the need for specific information, which plays an important role in their sociocultural groups, such as information about diet in the case of Chinese women with breast cancer [63]. Culturally tailored help with some specific characteristics of the disease could be added to improve the content of these apps. For instance, apps could help improve people’s perception of stigmatized diseases (such as cancer in some countries) and thus strengthen their engagement with their apps [63].

### The Need for Greater Personalization

One of the most critical factors stressed upon by many participants in these studies was the need to personalize the content of mHealth apps to a greater extent. Patients believed that since mHealth apps were created for their use, they should be able to personalize the apps to meet their own needs. From the patients’ point of view, using personalized apps could be as simple as changing the text size. It can be more complex to, for instance, make a drug regimen notification more flexible, give users the choice of adding personal information and receiving advice, or program a self-management app acting like a virtual personal coach [22,32,44,60]. Users wanted to be able to adjust the timing of their prompts, the number of symptoms reported, the frequency of health tips, and so forth [47]. Users also suggested that personalization of the app could also include the language used on the app: Some of them felt that the language used was rather patronizing, and they wanted to be able to change the type of language used to suit their tastes [47].

Greater personalization of apps could also make users feel more interactive with them. One user suggested, for example, that in order to meet health-related goals, they could have avatars that change, mature, and develop as the users learn more, achieve more goals, and become more independent by improving their self-management skills [34]. On the whole, patients value apps that are customized and tailored to meet their needs [25]. With more complex diseases such as cystic fibrosis, personalization is an essential requirement for users. With some diseases and conditions, management or treatment of the disease can vary from one patient to another, and patients may therefore have different routines to follow [51]. It would be best if these apps included “diverse functions” that enable patients to personalize and tailor them to meet their needs

### Accessibility Issues

In addition to the trustworthiness of the information and the appropriateness of the app, patients were also worried about a few issues related to their access to apps, such as the connectivity and cost. Some patients found that they had problems with their connection to these apps and other user interface issues. The frustration of not being able to connect with the apps was found to be particularly prevalent among older adults and the elderly, who frequently have poor eyesight and a lower level of digital literacy than other age groups [25,43,57,62]. The elderly patients included in the studies reviewed consisted of two groups: those wanting to acquire digital skills in order to be able to engage with these apps and those having no desire to improve their digital skills for this purpose [57]. Some expressed the feeling that placing greater reliance on technology meant that they were “admitting that the memory isn’t as good as it used to be,” which is distressing and prevented them from wanting to engage with their app [57]. Those with high-to-moderate levels of familiarity with computer technology were more likely to want to use apps, while those who were less familiar with computers and mobile phones were less likely to do so. People’s preferences for the use of apps varied, depending on their familiarity with this form of technology and their wish to become digitally competent. In addition to their digital incompetence, some patients have physical barriers that have to be to overcome to be able to use these apps. Elderly patients have described poor eyesight as one of the main barriers to using apps because they are not able to see their phone screens clearly [25,62].

Other accessibility challenges cited by patients are the extensive battery and memory requirements of smartphones [44]. For example, the mHealth app designed for pregnancy care used a lot of battery and memory space, which set patients problems. Patients had to ask themselves “what kind of apps do you delete?…[apps that use] lots of memory, lots of power, apps that are very active, [but] in that case your battery goes down…” [44]. Instead of being able to use their app to manage their care seamlessly, patients were bothered with having to think about whether an app was worth keeping.

Another issue patients faced was the cost of these apps [25], especially in developing countries where some patients cannot afford them [62]. In addition, some patients were worried about purchasing data to use the app when they were not living in an area with available Wi-Fi [62]. Not being able to use apps consistently because of data issues deterred some patients from engaging with them for long. An additional cost-related barrier was the fact that some apps charge patients a fee for obtaining full access to the app and for being advertisement-free [41,58]. Some patients would have to pay for using the full app, since some free versions provided only a few functions. The financial issues arising therefore prevented some potential app users from fully optimizing the use of an app and engaging with it.

## Discussion

### Principal Findings

To our knowledge, this is the first review of qualitative studies available in the literature that provides an overall picture of patients’ perceptions, beliefs, and experiences of mHealth apps. This study completes the model identifying the factors involved in the effective use of mHealth Apps for self-care purposes developed by Azhar and Dhillon [17]. In this model, the behavioral intention to use mHealth for self-care purposes is influenced by perceived usefulness, perceived ease of use, performance expectancy, social influence, self-efficacy, potential lack of privacy, and hedonic motives.

Our interpretation of qualitative findings shows how mHealth can strengthen patients’ engagement and sheds light on the dynamics of patients’ engagement and ways to make patients feel more empowered by using mHealth apps. As defined by Carman, the concept of patient engagement develops “as patients...and health professionals [work] in active partnership at various levels across the health care system - direct care, organizational design and governance, and policy making - to improve health and health care” [64]. Some authors have portrayed eHealth (internet and related technologies) as an important means of achieving patient engagement [65], especially in the case of isolated people and those who are hard to reach or have difficulty in remaining engaged in care [16]. Even when patients are not physically in a health care setting, health care advice and guidance are within easy reach at all times. From the patients’ point of view, mHealth could facilitate communication with health care providers and other patients, encourage them to be more participative during clinical encounters, and promote the use of coping techniques to manage their illness. However, some studies based on other methods have shown that some patients are reluctant to use mHealth apps, which is paradoxically the case with adolescent patients because they want to separate their feelings of being a patient from those of being a teenager and make their illnesses and diseases invisible [66]. In addition, they find apps, especially those with push notifications, annoying, intrusive, and time consuming [66]. Patients also stated that although these technical health innovations have supported them in many different respects, they still view their providers as the first point of contact to be consulted for discussing the options available. Health apps serve only as back-up consultations when they are really needed; apps are simply available to support physician-patient relationships and do not replace a physician in any way. mHealth is therefore not a substitute for care but simply a complementary tool [67].

Most of the studies included in this review suggest that patients feel empowered by the information provided by mHealth. WHO defines empowerment for health as: “…a process through which people gain greater control over decisions and actions affecting their life” [68]. According to the World Health Organization, adequate and understandable information is a necessary prerequisite for patient empowerment. Health care policymakers, politicians, and the media share the widespread idea that digital health technologies empower patients [9]. However, when faced with too much overwhelming information, users tend to feel more confused and possibly disempowered, which decreases the effectiveness of discussions with their physicians [67]. Users have also mentioned that the possible lack of validity of the information provided by apps makes it difficult to trust this information, which may make empowerment ambivalent. However, as mentioned above, if apps presented valid information more clearly and concisely, they could possibly incite users to engage more strongly with them as well as with users’ providers, caretakers, and support networks. Some authors have also reported that patients described themselves as reluctant, resistant, and anxious when using digital devices because they feel “disempowered” owing to the surveillance performed by some digital devices, which restricts their autonomy and reminds them that they are ill [69].

The results presented here suggest that despite the many advantages of mHealth apps, barriers to their successful adoption persist. Patients are still reluctant to rely solely on these tools for reasons related to privacy and security and the validity of the information provided. Other barriers to the optimal usage of apps are a lack of accessibility (the cost and absence of access to Wi-Fi) and issues concerning the technical and scientific validity of these tools. Many of the challenges could be met if there was more support on the part of health providers. In addition, standards could be developed and implemented to ensure that these apps provide patients with accurate evidence-based information. These standards could also address the security and privacy issues that many patients are concerned about as well as the compatibility of mobile apps with the technology with which existing health care systems are equipped. There is also a need for inexpensive quality apps and updates (possibly financed by health insurance funds or other agencies) that patients can easily afford.

The levels of engagement and empowerment resulting from the use of mobile phone apps and tools have been found to depend on the users. For example, one study showed that older adults were faced with barriers to adopting these tools because they were not as familiar with smartphones and tablets as younger people and had difficulty in using these technologies [70], whereas those who were digitally literate preferred to receive health information via tablets and electronic devices [70]. Patients’ requirements should be taken into account by those designing mobile health apps in order to alleviate some of the burden [71]. In addition, preference for the use of mobile devices can differ in some contexts. Female participants in Ghana spoke, for example, about how they did not comply voluntarily with health messages “because they didn’t see the point or because it went against their own experience or local knowledge” [72]. Users often expressed the feeling that automatic generic health messages seemed “depersonalized,” which may have resulted in “the opposite effects from those expected by the promoters of mHealth: Reduce caregiver-patient interactions and loosen the link with the health system” [72]. When developers create apps and tools of this kind for users, they should keep their users in mind and remember how their personal identity may influence users’ integration and engagement with the apps in order to facilitate adoption of the apps and the perpetuation of their use.

Although most authors focusing on developing countries discussed how mHealth apps help community health workers, few of them discussed the perspectives of patients using these apps. As the infrastructures with which the large cities in developing countries are improving, their inhabitants are gaining greater access to mobile data and Wi-Fi [73]. However, although the rates of penetration of mobile data and Wi-Fi have reached approximately 50% in these places, rural and underdeveloped regions still have no access at all to these services. It may be necessary to improve the infrastructure in developing countries’ rural and underdeveloped regions in order to be able to promote the use of mHealth apps [74].

### Limitations

Since only full-text articles available in English on PubMed were included in this review, many other studies have not been included because they were not written in English or were still in progress at the time of publication. The papers included here deal mainly with developed countries and less with developing countries, which limits the general validity of the results presented here. Since the rates of smartphone ownership in developing countries increased from 21% to 37% in 2015 and the active mobile phone subscription rates reached 53.6% in 2017, the uptake of mHealth apps may be different in these countries from the situation in more industrialized countries [3,75]. mHealth integration may have considerable implications in developing countries [76]. It is therefore worth noting the perceptions of individuals inhabiting developing countries in order to establish how this technological advancement is liable to improve or limit their access to health care [77].

### Conclusions

In this review, a meta-ethnographic approach was used to summarize the data published in 43 qualitative studies on patients’ perceptions of mHealth. Although mHealth apps were considered a useful complementary tool by many of the patients studied, some major issues emerged with regard to the optimal use of mHealth technologies, such as the need for more highly tailored designs, their cost, the validity of the information they provide, and issues such as privacy and security. Lastly, there is definitely a need for apps to be more personalized in order to meet the needs of individual users and their particular disease or condition, by designing apps that are easier to use, for example, by those who are not as digitally literate as others.

## Abbreviations

eHealth: electronic health
mHealth: mobile health
NOAC: Nonvitamin K antagonist oral anticoagulant
SMS: short message service
